# A Case of Opium Habit

**Published:** 1883-12

**Authors:** J. W. Putnam


### A Case of Opium Habit.

By J. W. Putnam, M. D.

Last February J. W., married lawyer, thirty-four years old, presented himself at the General Hospital for treatment.

He gave the following history: Six years ago, while in Texas, he was attacked with dysentery which was treated with opium. He took the drug for some time before he knew what had eased his pain.

On learning that it was opium, he tried to discontinue its use. It was too late; it had already become one of the necessaries of life. The history of the next six years is one of repeated trials to break the cursed habit. He would succeed in living without it for weeks, then a day of hard mental labor would leave him to toss all night on a sleepless pillow, arising in the morning weak and nervous, unable to do his day's work. "Then," he says, "I had to take opium to keep soul and body together."

This is his previous history, and to-day (Feb. 16th) he claims that he takes ten grains of morphine before breakfast and ten more on going to bed. His appetite is poor, his bowels move perhaps twice a week and he perspires but little. The tongue is dry and furred, skin rather yellow, pulse 88; temperature 98°; pupils not much contracted.

On questioning, patient says he is willing to do anything to be broken of this habit. I suggested to him the best way to stop was *to stop*. This he agreed to do, although he had taken ten grains sulph. morph. that morning. He was sent to the ward and ordered conc. tinct. avena sativa, ℳ xxx, every three hours, beef tea and capsicum at 11 A. M. and 4 P. M., in addition to the house diet.

Feb. 17th. Patient slept a little last night. He complains of a gnawing pain in his stomach, which he says is relieved about ten minutes after taking the avena sativa. His bowels moved twice to-day.

Feb. 18th. Slept three hours last night. To-day he feels weak and restless. Ordered whiskey, ℥ss., every three hours, and doubled the dose of avena (*i. e.*, ʒj). At 3 P. M. he suffered severe pain in bowels; has had diarrhœa all day; pulse 110, and weak. Ordered laudanum, ℥ss., per rectum. In two hours pulse fell to 90, the diarrhœa was checked and patient was comfortable.

Feb. 19th. Slept well last night, and feels easy this morning; appetite better than yesterday. Continued the treatment of avena and whiskey. There is considerable diaphoresis.

Feb. 20th. Patient complains that the avena sativa does not satisfy him, that he does not sleep. To me he seems stronger than yesterday; he has no diarrhœa and perspires much less. The appetite is not so good. Ordered fluid ext. coca, ʒj, every three hours, and pulv. arom., gr. xx, whenever he complained of the gnawing pain.

Feb. 22d. Passed a good night. Bowels moved twice yesterday. Appetite good to-day.

Feb. 28th. Stopped avena, coca, etc., and gave tinct. cinch. comp., a teaspoonful before meals, and tinct. ferri chlor., ♏ xx, after meals. He was also sent out with an attendant for exercise.

March 10th. Has been out every day this month, and his general appearance shows marked improvement. His walk is firmer, skin clearer and voice stronger. He sleeps well every night and his appetite improves steadily. He rarely complains of the gastric pain. A slight diarrhœa to-day, but it is easily checked with gallic acid, gr. x.

April 14th. Patient discharged. Recovered.

This case is of peculiar interest to me, as it shows the result of treatment by sudden withdrawal of the opium. From the date of admission, Feb. 16th, he had opium but once, on the 18th, and that, not because he craved or asked for the drug, but to check a severe diarrhœa. The avena sativa seemed, for a few days, to relieve the great distress in the stomach. The patient tells me that he never had taken anything which so nearly supplied the place of opium. This statement is difficult to understand, as I have never attributed to it any property save that of a bitter.

To-day, nearly nine months since he was admitted to the hospital, I was fortunate enough to meet him on the street. He was a different man. He had gained twenty-five pounds in weight, did a full day's work (out of door employment), and slept soundly at night. He tells me, and I believe him, that he has no desire for opium, and that he never would touch it again.

